# Green technology advancement, energy input share and carbon emission trend studies

**DOI:** 10.1038/s41598-024-51790-5

**Published:** 2024-01-23

**Authors:** YuXin Liu, Ping Lei, BingYang Shen, Dayi He

**Affiliations:** 1 School of Economics and Management, China University of Geosciences (BEIJING), Beijing, 100083 China; 2 Industrial Bank of China Limited, Fuzhou Chengbei Sub-branch, Fuzhou, 350003 Fujian Province China

**Keywords:** Environmental impact, Environmental economics, Sustainability

## Abstract

In order to study the theoretical mechanism of the impact of green technology progress on carbon emissions, this article constructs a theoretical mechanism of the impact of green technology progress on carbon emission growth. Explore the conditions for achieving carbon peak and carbon reduction. Based on the Cobb Douglas production function, construct a three sector model that includes capital, labor, and energy. Empirical methods were used to analyze the quantitative impact of green technology progress on carbon emission growth and the moderating effect of energy input share. This study mainly used provincial panel data from 1995 to 2020. Calculate carbon dioxide emissions based on energy consumption and carbon dioxide emission coefficients of various energy sources in different regions. Using the perpetual inventory method to calculate capital growth rate, green computing progress rate, etc., to provide data support for the green technology carbon reduction model. Empirical analysis of the impact of green technology progress on carbon emissions using the FGLS panel model. Theoretical and empirical analyses show that green technological progress promotes an increase in the carbon emission growth rate through the scale effect, with an impact coefficient of 0.607; it promotes a decrease in the carbon emission growth rate through the technological effect, with an impact coefficient of − 0.667; the combined effect promotes a decrease in growth rate of carbon emissions, with an impact coefficient of − 0.06. The share of energy inputs has a positive regulating effect on the scale effect.

## Introduction

The strategies of carbon peaking and carbon neutrality are the inevitable requirements for implementing the new development concept, constructing a new development pattern, and promoting green and low-carbon transformation, while promoting sustained economic growth is the long-term goal of China as a developing country and an intrinsic requirement for achieving high-quality development. To achieve the unity of sustained economic growth and carbon emission reduction, green technology progress will become the key "engine" to solve this problem^1^. Green technological progress can promote economic growth and effectively control and reduce fossil fuel emissions at the same time, thus promoting energy conservation and carbon emission reduction by enterprises^2–4^. The empirical facts of countries worldwide show that green technology plays a very important role in the process of carbon emission changes and economic growth. This leads us to think: how do the rate of green technology progress and the growth rate of carbon emissions interact with each other? How can carbon emissions peak and become carbon neutral under the influence of the rate of progress of green technologies? What other factors influence the interaction between the rate of green technology progress and the rate of carbon emission growth? These questions are new developments in similar studies and are central questions that require an answer in the study of green technological progress and carbon emissions.

The empirical data on China's green technology progress rate and carbon emission growth rate are further analysed below.

**Figure 1:**
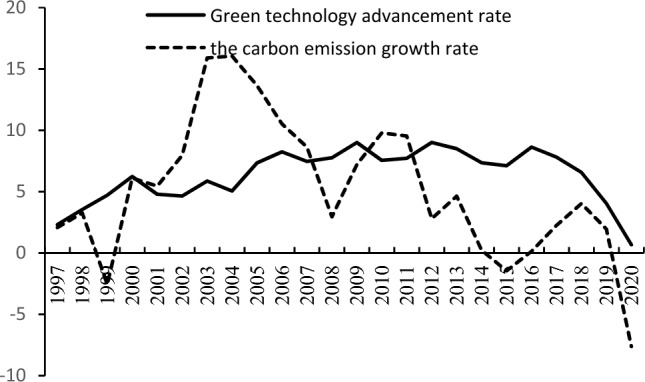
China's Green Technology Growth Rate and Carbon Emission Growth Rate (1997–2020).

As shown in Fig. 1, the growth rate of carbon emissions peaked in approximately 2004 and has been declining since then. Carbon emissions will continue to grow until 2020 and start to decline after 2020, which means that they will not continue to grow but will eventually peak and decline; the growth rate of green technology peaked at approximately 2013, after which the overall trend has been declining, but the level of green technology has been in a state of growth.

**Figure 2:**
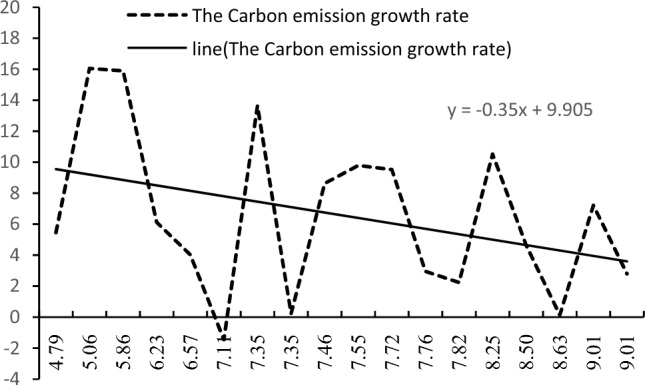
Trend of the Carbon Emission Growth Rate with China's Green Technology Growth Rate (1997–2020).

Figure 2 gives a graph of the trend of the growth rate of carbon emissions in China with the change in the rate of progress of green technology. As the green technology progress rate increases, the carbon emission growth rate shows fluctuating changes, but the overall trend is decreasing. As shown in the trend line, the carbon emission growth rate is linearly negatively correlated with the green technology progress rate. Therefore, there is a negative correlation between the rate of progress in green technology and the growth rate of emissions.

From the above empirical data, the following empirical facts can be observed about the rate of progress of green technology and the growth rate of carbon emissions in China: (1) in the process of economic growth, green technology continues to progress, but the rate of growth gradually decreases; (2) the growth rate of carbon emissions is in a declining trend in general, and finally decreases to a negative level, as the carbon emissions first increase and then decrease, showing an inverted "U"-shaped trend; (3) the rate of progress of green technology and the overall rate of growth of carbon emissions have a negative correlation.

Based on the literature, Green technologies have a significant impact on carbon emissions. Green technologies affect carbon emissions mainly through channels that influence energy efficiency, resource allocation and the structure of energy consumption^3,5,6^. Some scholars believe that green technological innovation is beneficial to carbon emission reduction^7,8^. Meirun et al.^7^ found that there is a significant negative correlation between green technological innovation and carbon emissions in both the long and short term. Wu and Zhao^8^ found that there is a spatial spillover effect of green technology. Improvement in the level of green technology progress in local or neighbouring areas has a promotional effect on local energy conservation and emission reduction.Some studies concluded that green technology progress has a significant negative impact on carbon emissions^7,9^. The study found that in more economically developed provinces, carbon emissions increase as the level of green technology develops. This is due to the “rebound effect”^10,11^. Less literature focuses on the impact of the green technology growth rate on the carbon emission growth rate^12^, and less literature investigates the mechanism by which energy input elasticity and carbon emission elasticity play a role in this impact. Due to the differences in development stages and types of firms, less literature focuses on the differences in the impact of green technology progress on different types of firms.

Based on the established research background, this paper tries to compensate for the above research deficiencies. First, based on Aghion et al.^13^, this paper constructs a three-sector production model including capital, labour and energy, gives the theoretical mechanism of the impact of the green technology progress rate on the growth rate of carbon emissions, and discusses the conditions for achieving carbon peaking and carbon emission reduction. Second, based on the theoretical conclusions, this paper conducts empirical analyses to study the quantitative impact of the growth rate of green technology on the growth rate of carbon emissions and the moderating role of the share of energy inputs.

The innovations of this paper are mainly in the following two aspects:

First, to construct a new endogenous growth model that includes green technological progress. Based on the existing theory of endogenous growth of the environment, green technological progress will be included in it. And on this basis, analyse the dynamic relationship between the coefficient of green technological progress rate and carbon dioxide emission rate. The case of carbon peaking is also analysed in the theoretical model.

Second, Exploring new mechanisms for carbon emission reduction through green technological advances.The moderating role of energy input shares is further discussed.

The main significance of the article. By examining the impact of green technology progress on carbon emissions and its mechanism of action, the mechanism of green technology progress on carbon emissions is further clarified. It provides an effective basis for playing the role of green technology in the successful realisation of carbon peak in China.

The theoretical conclusions of this paper show that green technology progress promotes the increase in the growth rate of carbon emissions through the scale effect, given the continuous improvement of the rate of green technology progress; green technology progress promotes the reduction of the growth rate of carbon emissions through the technological effect. The technological effect of the rate of green technology progress on the impact of the growth rate of carbon emissions is greater than the scale effect, ultimately affecting the promotion of the growth rate of carbon emissions as it continues to decrease. When the growth rate of carbon emissions is 0, a carbon peak is achieved; when the growth rate of carbon emissions is reduced to negative, carbon emission reduction is achieved. In addition, the share of energy inputs has a positive moderating effect on the scale effect.

The empirical analysis of this paper shows that the rate of progress of the green technology level has a significant negative impact on the growth rate of carbon emissions, and the impact coefficient is approximately − 0.06, which is consistent with theoretical proposition 3. The green technology progress rate impact on the carbon emissions growth rate is significantly negative and the mechanism is the technology effect, with the impact coefficient at − 0.667; based on this, we can calculate the scale effect of the impact coefficient of 0.607 and theoretical proposition 2 to maintain consistency. Through the moderating effect test, it is found that the moderating effect of the energy input share on the scale effect is significantly positive, and hence it is consistent with theoretical proposition 1. Finally, the paper conducts robustness and heterogeneity analyses.

The remaining chapter is organised as follows: “Theory/calculation” Section Material and methods, presents the theoretical model construction and analysis to derive the theoretical mechanism of the impact of the green technology progress rate on the growth rate of carbon emissions; “Material and methods” Section Theory/calculation and results, presents the empirical analysis and the construction of an econometric model to investigate the empirical impact of the green technology progress rate on the growth rate of carbon emissions, it conducts robustness and heterogeneity analyses. “Analysis of the empirical results” Section outlines the conclusions and policy recommendations.

## Theory/calculation

### Theoretical modelling

This section builds on Aghion et al.^13^ to analyse the impact of green technological progress on carbon emissions by constructing a three-sector production model incorporating capital, labour and energy. It is assumed that the firm’s production function takes the C-D form, denoted as:

1 $$Y_{t} = (A_{t} L_{t} )^{1 - \alpha - \beta } (B_{t} K_{t} )^{\beta } (\delta_{t} E_{t} )^{\alpha }$$

In the above equation, $Y_{t}$ represents the output of the final product, $K_{{\text{t}}}$ denotes capital inputs, $L_{t}$ denotes labour inputs, $E_{t}$ denotes energy inputs. The subscript t denotes the time, $A_{t}$ indicates labour-saving technology level, $B_{t}$ represents the technological level of capital factors, $A_{t}$ and $B_{t}$ are collectively referred to as the level of technology (without green technology), and $\delta_{t}$ denotes the level of green technology. Green technologies here denote improvements in energy efficiency. The parameters $\alpha$ and $\beta$ represent the shares of energy inputs and capital inputs in the production function, respectively, with $0 < \alpha < 1$ and $0 < \beta < 1$. The energy input share $\alpha$ represents the amount of energy invested in the production process and reflects the extent to which the economy consumes energy. A higher energy input share $\alpha$ indicates a higher degree of energy consumption.

Drawing on Shen et al.^12^, it is assumed that the production of one unit of energy requires one unit of final product inputs and that the price of the final product is normalised to 1. The price of energy in the energy market is $p_{e}$, the price of labour in the labour market is $p_{l}$, and the price of capital in the capital market is $p_{k}$. Assuming that the final product market is a perfectly competitive market, according to the firm's profit maximisation condition:

2 $$\mathop {{\text{max}}}\limits_{{K_{t} ,L_{t} ,E_{t} }} (A_{t} L_{t} )^{1 - \alpha - \beta } (B_{t} K_{t} )^{\beta } (\delta_{t} E_{t} )^{\alpha } - p_{kt} K_{t} - p_{lt} L_{t} - p_{et} E_{t}$$

The above equation takes a first-order derivative of $E_{t}$. Further collation gives the demand function for energy $E_{t}$ as:

3 $$E_{t} = \left[ {\frac{{\alpha (A_{t} L_{t} )^{1 - \alpha - \beta } (B_{t} K_{t} )^{\beta } \delta_{t}^{\alpha } }}{{P_{et} }}} \right]^{{\frac{1}{1 - \alpha }}}$$

From the above equation, the elasticity of demand for energy can be calculated as a constant $\frac{1}{1 - \alpha }$. The profit maximisation function of the energy producer is:

4 $$\mathop {MAX}\limits_{{P_{et} }} P_{et} E_{t} - E_{t}$$

Solving the first-order condition for maximisation of the above equation gives:

5 $$- \alpha P_{et}^{{\frac{ - 1}{{1 - \alpha }}}} + P_{et}^{{\frac{ - 2 + \alpha }{{1 - \alpha }}}} = 0 \, \Rightarrow \, P_{et} = \frac{1}{\alpha }$$

Therefore, the price of energy in the energy market is $\frac{1}{\alpha }$. Substituting into Eq. (3) gives the energy demand function as:

6 $$E_{t} = \alpha^{{\frac{2}{1 - \alpha }}} [(A_{t} L_{t} )^{1 - \alpha - \beta } (B_{t} K_{t} )^{\beta } ]^{{\frac{1}{1 - \alpha }}} \delta_{t}^{{\frac{\alpha }{1 - \alpha }}}$$

To facilitate the portrayal of the two-way relationship between energy inputs and economic output, as outlined in Kumbaroğlu et al.^14^, gross domestic product (GDP) is expressed as the difference between output and energy costs:

7 $$\begin{aligned} GDP_{t} = & Y_{t} - p_{et} E_{t} \\ { = } & (A_{t} L_{t} )^{1 - \alpha - \beta } (B_{t} K_{t} )^{\beta } (\delta_{t} E_{t} )^{\alpha } - \frac{1}{\alpha }E_{t} \\ { = } & \alpha^{{\frac{2\alpha }{{1 - \alpha }}}} (1 - \alpha )[(A_{t} L_{t} )^{1 - \alpha - \beta } (B_{t} K_{t} )^{\beta } ]^{{\frac{1}{1 - \alpha }}} \delta_{t}^{{^{{\frac{\alpha }{1 - \alpha }}} }} \\ \end{aligned}$$

Drawing on Lin and Li^15^, assuming that carbon emissions come only from the energy use process, the expression for carbon emissions is as follows:

8 $$D_{t} = \delta_{t}^{ - \lambda } E_{t}$$

In the above equation, $D_{t}$ represents carbon emissions, $\delta_{t}^{ - \lambda }$ measures the technological effect of the level of green technology on CO2 emissions, $\lambda$ denotes the elasticity of the impact of the level of green technology on carbon emissions, and $\lambda > 0$ indicating that the higher the carbon emissions of green technology, the smaller the direct carbon emissions, i.e., the technology effect of the level of green technology on CO2 emissions is negative.

### Theoretical model analysis

Joining (6) and (8), the total carbon emissions can be obtained as follows:

9 $$D_{t} = \alpha^{{\frac{2}{1 - \alpha }}} \left[ {(A_{t} L_{t} )^{1 - \alpha - \beta } (B_{t} K_{t} )^{\beta } } \right]^{{\frac{1}{1 - \alpha }}} \delta_{t}^{{\frac{\alpha }{1 - \alpha } - \lambda }}$$

Joining Eqs. (7) and (9) gives:

10 $$D_{t} = GDP_{t} *\alpha^{2} (1 - \alpha )^{ - 1} \delta_{t}^{ - \lambda }$$

From the above equation, the total carbon emissions are determined by the total economy (GDP), the share of energy inputs ($\alpha$), the level of green technology ($\delta_{t}$) and the carbon elasticity of the level of green technology ($\lambda$).

Taking the natural logarithm of both sides of Eq. (7) and deriving the GDP growth rate $\left( {\frac{{\mathop {GDP}\limits^{ \cdot } }}{GDP}} \right)$, the growth rate of the variable is equal to the rate of change of its natural logarithm) for both the left and right sides simultaneously, is:

11 $$\frac{{\mathop {GDP}\limits^{ \cdot } }}{GDP}{ = }\frac{1 - \alpha - \beta }{{1 - \alpha }}\left( {\frac{{\dot{A}}}{A} + \frac{{\dot{L}}}{L}} \right) + \frac{\beta }{1 - \alpha }\left( {\frac{{\dot{B}}}{B} + \frac{{\dot{K}}}{K}} \right) + \frac{\alpha }{1 - \alpha }\frac{{\dot{\delta }}}{\delta }$$

Similarly, taking the natural logarithm on both sides of Eq. (10), further derivation leads to the growth rate of carbon emissions as follows:

12 $$\frac{{\dot{D}}}{D} = \frac{{\mathop {GDP}\limits^{ \cdot } }}{GDP} - \lambda \frac{{\dot{\delta }}}{\delta }$$

Joining Eqs. (11) and (12), and organising, one obtains:

13 $$\frac{{\dot{D}}}{D} = \left( {\frac{\alpha }{1 - \alpha } - \lambda } \right)\frac{{\dot{\delta }}}{\delta } + \frac{1 - \alpha - \beta }{{1 - \alpha }}\left( {\frac{{\dot{A}}}{A} + \frac{{\dot{L}}}{L}} \right) + \frac{\beta }{1 - \alpha }\left( {\frac{{\dot{B}}}{B} + \frac{{\dot{K}}}{K}} \right)$$

In Eq. (13), $\frac{\alpha }{1 - \alpha } - \lambda$ is the coefficient of the impact of the rate of green technological progress on the growth rate of carbon emissions, which measures the comprehensive impact of the rate of green technological progress on the growth rate of carbon emissions.

In the following, the theoretical mechanism by which the rate of green technology progress affects the growth rate of carbon emissions is further analysed.

Equation (11) is obtained by taking the derivative of $\frac{{\mathop {GDP}\limits^{ \cdot } }}{GDP}$ with respect to $\frac{{\dot{\delta }}}{\delta }$:

14 $${{\partial \left( {\frac{{\mathop {GDP}\limits^{ \cdot } }}{GDP}} \right)} \mathord{\left/ {\vphantom {{\partial \left( {\frac{{\mathop {GDP}\limits^{ \cdot } }}{GDP}} \right)} {\partial \left( {\frac{{\dot{\delta }}}{\delta }} \right)}}} \right. \kern-0pt} {\partial \left( {\frac{{\dot{\delta }}}{\delta }} \right)}}{ = }\frac{\alpha }{1 - \alpha } > 0$$

In the above equation, $\frac{\alpha }{1 - \alpha }$ represents the coefficient of influence of green technology progress rate on GDP growth rate. The larger the share of energy inputs $\alpha$, the larger the impact coefficient $\frac{\alpha }{1 - \alpha }$.

(12) in which $\frac{{\dot{D}}}{D}$ is derived with respect to $\frac{{\mathop {GDP}\limits^{ \cdot } }}{GDP}$ avails the following:

15 $${{\partial \left( {\frac{{\dot{D}}}{D}} \right)} \mathord{\left/ {\vphantom {{\partial \left( {\frac{{\dot{D}}}{D}} \right)} {\partial \left( {\frac{{\mathop {GDP}\limits^{ \cdot } }}{GDP}} \right)}}} \right. \kern-0pt} {\partial \left( {\frac{{\mathop {GDP}\limits^{ \cdot } }}{GDP}} \right)}}{ = 1}$$

The above equation indicates that the GDP growth rate has a coefficient of influence on the growth rate of carbon emissions of 1. By associating Eqs. (14) and (15), and by taking the derivative of $\frac{{\dot{D}}}{D}$ with respect to $\frac{{\dot{\delta }}}{\delta }$, the following is obtained:

16 $$\partial \left( {\frac{{\dot{D}}}{D}} \right){/}\partial \left( {\frac{{\dot{\delta }}}{\delta }} \right){ = }\partial \left( {\frac{{\dot{D}}}{D}} \right)/\partial \left( {\frac{{\mathop {GDP}\limits^{ \cdot } }}{GDP}} \right) \cdot \left[ {{{\partial \left( {\frac{{\mathop {GDP}\limits^{ \cdot } }}{GDP}} \right)} \mathord{\left/ {\vphantom {{\partial \left( {\frac{{\mathop {GDP}\limits^{ \cdot } }}{GDP}} \right)} {\partial \left( {\frac{{\dot{\delta }}}{\delta }} \right)}}} \right. \kern-0pt} {\partial \left( {\frac{{\dot{\delta }}}{\delta }} \right)}}} \right] = 1 \cdot \frac{\alpha }{1 - \alpha } = \frac{\alpha }{1 - \alpha }$$

The above equation represents the impact coefficient of the green technology progress rate indirectly affecting the growth rate of carbon emissions by influencing the size of GDP, with the coefficient $\frac{\alpha }{1 - \alpha }$. Green technological advances lead the way in driving economic scale growth, thus driving further increases in the rate of growth of carbon emissions, a theoretical mechanism we call the scale effect. Since $\alpha > 0$, $\frac{\alpha }{1 - \alpha } > 0$, i.e., the scale effect is positive, the economic implication of the scale effect is that an increase in the rate of green technological progress results in an increasing rate of growth of the total economy ($GDP$), hence driving an increase in the rate of growth of carbon emissions.

Equation (16) shows that the size of the scale effect depends on the energy input share $\alpha$, thereby the larger $\alpha$ is, the larger the scale effect is. Therefore, the energy input share has a positive moderating effect on the scale effect, leading to the following theoretical proposition:

#### Proposition 1

Energy input share $\alpha$ positively moderates the scale effect.

The economic implication of Proposition 1 is that an increase in the share of energy inputs promotes an increase in the scale effect, which in turn drives up the growth rate of carbon emissions. In fact, the share of energy inputs $\alpha$ in the production process varies across economies due to differences in industrial structure, energy endowment, etc., and hence differences in scale effects. The higher the share of energy inputs $\alpha$, i.e., the more energy-input-dependent economic production is, the larger the scale effect of the rate of green technological progress and the higher the rate of growth of carbon emissions.

Equation (12) in which $\frac{{\dot{D}}}{D}$ is derived with respect to $\frac{{\dot{\delta }}}{\delta }$, thus obtaining:

17 $${{\partial \left( {\frac{{\dot{D}}}{D}} \right)} \mathord{\left/ {\vphantom {{\partial \left( {\frac{{\dot{D}}}{D}} \right)} {\partial \left( {\frac{{\dot{\delta }}}{\delta }} \right)}}} \right. \kern-0pt} {\partial \left( {\frac{{\dot{\delta }}}{\delta }} \right)}} = - \lambda$$

In the above equation, $- \lambda$ represents the direct impact coefficient of the green technology progress rate on the carbon emission growth rate, which measures the technological impact of green technology progress on the carbon emission growth rate. In this case, the increase in the rate of progress of green technology directly contributes to the reduction of the growth rate of carbon emissions, and this theoretical mechanism is called the technology effect. Since $\lambda > 0$ and therefore $- \lambda < 0$, the technology effect is negative. The economic meaning of the technology effect is that the rate of green technological progress contributes to a decrease in the rate of growth of carbon emissions directly through technological progress.

The combined effect of the rate of green technological progress on the growth rate of carbon emissions depends on the combined effect of the scale and technology effects of the rate of green technological progress. Equation (13) by taking the derivative of $\frac{{\dot{D}}}{D}$ with respect to $\frac{{\dot{\delta }}}{\delta }$ in Eq, the following is obtained:

18 $${{\partial \left( {\frac{{\dot{D}}}{D}} \right)} \mathord{\left/ {\vphantom {{\partial \left( {\frac{{\dot{D}}}{D}} \right)} {\partial \left( {\frac{{\dot{\delta }}}{\delta }} \right)}}} \right. \kern-0pt} {\partial \left( {\frac{{\dot{\delta }}}{\delta }} \right)}} = \frac{\alpha }{1 - \alpha } - \lambda$$

The above equation represents the combined effect of the rate of green technology progress on the growth rate of carbon emissions, which leads to theoretical proposition 2:

#### Proposition 2

As the rate of green technological progress continues to increase, green technological progress increases the growth rate of carbon emissions through the scale effect, with an impact coefficient of $\frac{\alpha }{1 - \alpha } > 0$. Green technological progress contributes to a reduction in the growth rate of carbon emissions through the technology effect, with an impact factor of $- \lambda < 0$. The combined effect coefficient of the rate of green technological progress on the growth rate of carbon emissions is $\frac{\alpha }{1 - \alpha } - \lambda$.

Proposition 2 gives the theoretical mechanism of the impact of the rate of progress of green technology on the growth rate of carbon emissions. The economic implication of Proposition 2 is that when the rate of progress of green technology increases, on the one hand, technological progress promotes the growth of economic scale (GDP), further promoting the increase in the growth rate of carbon emissions; on the other hand, green technology directly promotes the reduction in the growth rate of carbon emissions, and the combined effect depends on the relative size of the two.

**Figure 3:**
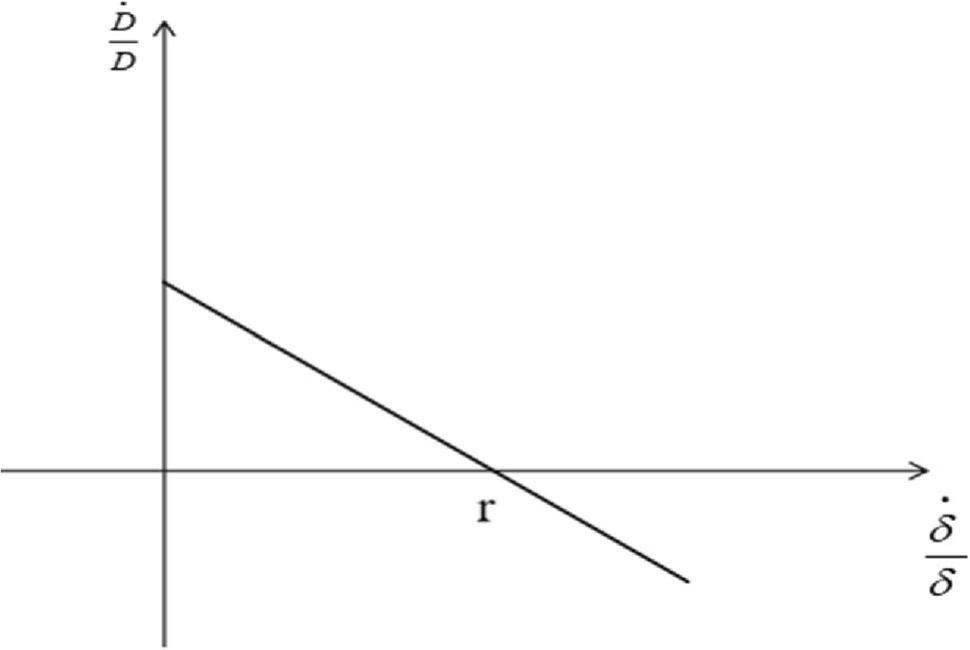
Impact of the rate of green technological progress on the growth rate of carbon emissions $\left( {\frac{\alpha }{1 - \alpha } - \lambda < 0} \right)$.

Different scenarios of the relative magnitude of scale and technology effects are considered below:

When $\frac{\alpha }{1 - \alpha } - \lambda < 0$, the scale effect of green technological progress is smaller than the technological effect, and the growth rate of carbon emissions keeps decreasing when the rate of green technological progress increases, i.e. $\left( {\frac{\alpha }{1 - \alpha } - \lambda } \right)\frac{{\dot{\delta }}}{\delta } < 0$, which is consistent with the empirical facts. Since $\frac{1 - \alpha - \beta }{{1 - \alpha }}\left( {\frac{{\dot{A}}}{A} + \frac{{\dot{L}}}{L}} \right) + \frac{\beta }{1 - \alpha }\left( {\frac{{\dot{B}}}{B} + \frac{{\dot{K}}}{K}} \right) > 0$, the growth rate of carbon emissions, $\frac{{\dot{D}}}{D}$, decreases as the rate of green technological progress, $\frac{{\dot{\delta }}}{\delta }$, continues to increase. When the growth rate of carbon emissions $\frac{{\dot{D}}}{D} < 0$, it means that carbon emissions start to decrease continuously; when the growth rate of carbon emissions $\frac{{\dot{D}}}{D} = 0$, carbon emissions reach the maximum peak. Figure 3 shows the correlation between the growth rate of carbon emissions $\left( {\frac{{\dot{D}}}{D}} \right)$ and the rate of green technology progress $\left( {\frac{{\dot{\delta }}}{\delta }} \right)$ in this case.

**Figure 4:**
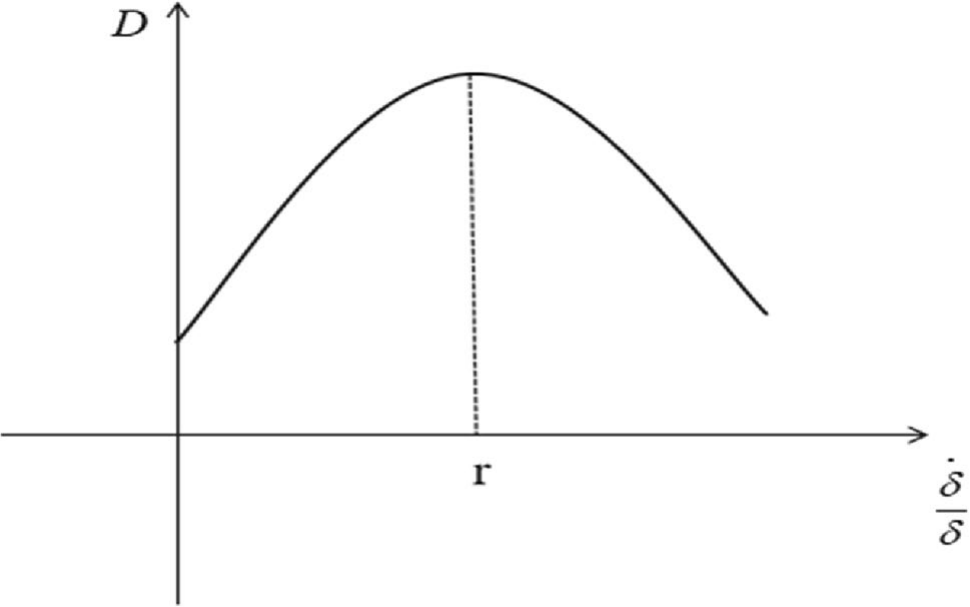
Impact of the green technology progress rate on carbon emissions $\left( {\frac{\alpha }{1 - \alpha } - \lambda < 0} \right)$.

As shown in Fig. 4, the carbon emissions at this point can be expressed as:

As shown in Fig. 4, carbon emissions first increase and then decrease with the progress of green technology. When the growth rate of carbon emissions x = 0, the carbon peak is achieved, and thereafter, as the level of green technology is further improved, the growth rate of carbon emissions drops to a negative value, and carbon emissions continue to decrease.

From Eq. (13), when carbon peaking is achieved, it needs to be satisfied:

19 $$\frac{{\dot{D}}}{D}{ = }\frac{1 - \alpha - \beta }{{1 - \alpha }}\left( {\frac{{\dot{A}}}{A} + \frac{{\dot{L}}}{L}} \right) + \frac{\beta }{1 - \alpha }\left( {\frac{{\dot{B}}}{B} + \frac{{\dot{K}}}{K}} \right) + \left( {\frac{\alpha }{1 - \alpha } - \lambda } \right)\frac{{\dot{\delta }}}{\delta } = 0$$

At this point, the direct effect of green technological progress, i.e., the reduction in the rate of growth of carbon emissions facilitated by green technological progress, is offset by the increase in the rate of growth of carbon emissions brought about by economic growth, and carbon peaking is achieved. At peak carbon, the level of green technology can be expressed as:

20 $$\frac{{\dot{\delta }}}{\delta } = {{\left[ {\frac{1 - \alpha - \beta }{{1 - \alpha }}\left( {\frac{{\dot{A}}}{A} + \frac{{\dot{L}}}{L}} \right) + \frac{\beta }{1 - \alpha }\left( {\frac{{\dot{B}}}{B} + \frac{{\dot{K}}}{K}} \right)} \right]} \mathord{\left/ {\vphantom {{\left[ {\frac{1 - \alpha - \beta }{{1 - \alpha }}\left( {\frac{{\dot{A}}}{A} + \frac{{\dot{L}}}{L}} \right) + \frac{\beta }{1 - \alpha }\left( {\frac{{\dot{B}}}{B} + \frac{{\dot{K}}}{K}} \right)} \right]} {\left( {\lambda - \frac{\alpha }{1 - \alpha }} \right)}}} \right. \kern-0pt} {\left( {\lambda - \frac{\alpha }{1 - \alpha }} \right)}}$$

The combined analysis of the two cases and the empirical facts show that $\frac{\alpha }{1 - \alpha } - \lambda < 0$ holds, thus leading to theoretical proposition 3:

#### Proposition 3

The technological effect of the rate of green technological progress on the growth rate of carbon emissions is greater than the scale effect, thus driving the growth rate of carbon emissions to decrease. When the growth rate of carbon emissions is 0, a carbon peak is achieved; when the growth rate of carbon emissions is reduced to negative, carbon emission reduction is achieved.

The economic meaning of proposition 3 is that the final impact of the green technology progress rate on the growth rate of carbon emissions is negative; that is, the green technology progress rate to promote the growth rate of carbon emissions continues to decline. It can be seen from formula (19) that when the rate of green technology progress drives the growth rate of carbon emissions to reduce to 0, a carbon peak is achieved; when the growth rate of carbon emissions is reduced to negative, carbon emissions begin to decrease, and carbon emission reduction is achieved. Proposition 3 explains the inverted “U”-shaped trend of carbon emissions in the empirical facts (as shown in Fig. 4), providing a theoretical basis for carbon peak and carbon neutrality from the perspective of the rate of progress of green technology.

## Material and methods

### Modelling

This section constructs an econometric model to test propositions 3 derived from the theoretical model and empirically analyses the scale effect $\left( {\frac{\alpha }{1 - \alpha }} \right)$, the technology effect ($- \lambda$), and the combined effect $\left( {\frac{\alpha }{1 - \alpha } - \lambda } \right)$ of the rate of green technological progress on the rate of growth of carbon emissions, using empirical data. In addition, the moderating role of energy input share $\alpha$ on the scale effect is considered. This section rewrites Eq. (13) as follows:

21 $$\frac{{\dot{D}_{it} }}{{D_{it} }}{ = }\alpha_{1} \frac{{\dot{\delta }_{it} }}{{\delta_{it} }} + \alpha_{2} \frac{{\dot{L}_{it} }}{{L_{it} }} + \alpha_{3} \frac{{\dot{K}_{it} }}{{K_{it} }} + \alpha_{4} \frac{{\dot{\varphi }_{it} }}{{\varphi_{it} }} + \alpha_{5} \chi_{it} + \mu_{i} + \omega_{t} + \varepsilon_{it}$$

where $\frac{{\dot{D}_{it} }}{{D_{it} }}$ denotes the growth rate of carbon emissions in region i in year t. $\frac{{\dot{\delta }_{it} }}{{\delta_{it} }}$ denotes the rate of green technology progress in region i in year t.$\alpha_{1} = \frac{\alpha }{1 - \alpha } - \lambda$ represents the comprehensive influence coefficient of the green technology progress rate on the growth rate of carbon emissions. If $\alpha_{1} < 0$, then proposition 3 holds. Therefore, the growth rate of carbon emissions is reduced with the increase in the rate of progress of green technology, carbon emissions increase and then decrease, and there is a carbon peak situation. The coefficient of $\frac{{\dot{\delta }_{it} }}{{\delta_{it} }}$ can be obtained from the regression to obtain the specific value of the combined effect of the rate of green technological progress on the growth rate of carbon emissions, $\frac{\alpha }{1 - \alpha } - \lambda$. $\alpha_{2} = \frac{1 - \alpha - \beta }{{1 - \alpha }}$ denotes the coefficient on the labour growth rate, $\frac{{\dot{L}_{it} }}{{L_{it} }}$ denotes the labour growth rate, $\alpha_{3} = \frac{\beta }{1 - \alpha }$ denotes the coefficient on the capital growth rate, $\frac{{\dot{K}_{it} }}{{K_{it} }}$ denotes the capital growth rate, $\alpha_{4} \frac{{\dot{\varphi }_{it} }}{{\varphi_{it} }} = \left( {\frac{1 - \alpha - \beta }{{1 - \alpha }}\frac{{\dot{A}}}{A} + \frac{\beta }{1 - \alpha }\frac{{\dot{B}}}{B}} \right)$ denotes the effect of the rate of labour-and capital-related technological progress, $\alpha_{5} \chi_{it}$ denotes the other control variables and their coefficients, $\mu_{i}$ denotes the individual fixed effects, $\omega_{t}$ denotes the time-fixed effects, and $\varepsilon_{it}$ denotes the error term.

The moderating role of energy input share $\alpha$ on the scale effect is investigated below by rewriting Eq. (13) as follows:

22 $$\frac{{\dot{D}_{it} }}{{D_{it} }} \, = \beta_{1} \frac{{\dot{\delta }_{it} }}{{\delta_{it} }} + \beta_{2} W_{it} \cdot \frac{{\dot{\delta }_{it} }}{{\delta_{it} }} + \beta_{3} W_{it} + \alpha_{2} \frac{{\dot{L}_{it} }}{{L_{it} }} + \alpha_{3} \frac{{\dot{K}_{it} }}{{K_{it} }} + \alpha_{4} \frac{{\dot{\varphi }_{it} }}{{\varphi_{it} }} + \alpha_{5} \chi_{it} + \mu_{i} + \omega_{t} + \varepsilon_{it}$$

In the above formula, the coefficient $\beta_{1} = - \lambda$ indicates the technical effect of the green technology progress rate on the growth rate of carbon emissions, which reflects the part of the coefficient of influence of the green technology progress rate on the growth rate of carbon emissions that has nothing to do with the share of energy inputs, and hence if the regression coefficient $\beta_{1} < 0$, the technological effect of promoting the reduction of the growth rate of carbon emissions in proposition 2 is established; $W_{{{\text{it}}}}$ denotes the energy input share $\alpha$ of different economies, where the energy input share is treated as a variable that varies over time and within the economy, and also $W_{{{\text{it}}}}$ is equivalent to the variable form of $\alpha$; $W_{it} \cdot \frac{{\dot{\delta }_{it} }}{{\delta_{it} }}$ is the interaction term between the energy input share and the rate of green technological progress.$W_{{{\text{it}}}}$ can be interpreted as a moderator variable for the coefficient of the impact of the rate of green technological progress $\left( {\frac{{\dot{\delta }_{it} }}{{\delta_{it} }}} \right)$ on the growth rate of carbon emissions $\left( {\frac{{\dot{D}_{it} }}{{D_{it} }} \, } \right)$; $\beta_{2}$ denotes the coefficient of the interaction term; and $\beta_{3}$ denotes the coefficient of the share of energy inputs. Since the scale effect $\frac{\alpha }{1 - \alpha }$ is positively correlated with respect to the energy input share $\alpha$, Proposition 1 holds if the coefficient $\beta_{2}$ is significantly positive.

### Selection of indicators

#### Explained variable

Carbon emission growth rate $\left( {\frac{{\dot{D}_{it} }}{{D_{it} }} \, } \right)$. Carbon emissions are measured by carbon dioxide emissions, and according to the research practice^12,16^, this paper uses the carbon dioxide emissions estimation reference method of the United Nations Intergovernmental Panel on Climate Change (IPCC) for the estimation of carbon emissions, and the consumption of various energy sources, such as coal, coke, crude oil, etc., is used as a baseline for the conversion estimation^17,18^. The growth rate of carbon emissions in 30 provinces, municipalities and autonomous regions in China from 1995 to 2020 can be calculated.

#### Core explanatory variable

Green technology progress rate $\left( {\frac{{\dot{\delta }_{it} }}{{\delta_{it} }}} \right)$. Drawing on Wu^19^, the level of green technology is measured by the number of green technology patents, and using the perpetual inventory method, the rate of green technology progress in China's provinces, municipalities and autonomous regions from 1995 to 2020 can be calculated.

#### Moderator variable

Energy input share ($W_{{{\text{it}}}}$). Drawing on Yi et al.^5^, since the energy input share is usually positively correlated with coal use as a share of total energy, this paper measures the energy input share as the ratio of coal use to total energy (million tonnes of marked coal).

#### Other explanatory variables

(i) Capital growth rate $\left( {\frac{{\dot{K}_{it} }}{{K_{it} }}} \right)$, drawing on (Chen et al. 2020)^20^, and it is calculated by the perpetual inventory method. (ii) Labour growth rate $\left( {\frac{{\dot{L}_{it} }}{{L_{it} }}} \right)$, expressed as the growth rate of employees in each province, city and autonomous region from 1995 to 2020, according to Cheng et al.^21^. (iii) The technology growth rate of capital and labour, referred to as the technology growth rate $\left( {\frac{{\dot{\varphi }_{it} }}{{\varphi_{it} }}} \right)$, is measured by the total factor productivity growth rate, drawing on Luo and Zhang^22^.

#### Control variables

(i) Level of government intervention, measured by the share of local government fiscal expenditure in regional GDP (Wang et al. 2020)^23^. (ii) The degree of marketisation. The market plays a major role in the process of resource allocation, and the rational allocation of resources has a positive effect on carbon emission reduction. The degree of marketisation is measured by the FAN marketisation index (Wang and Chen 2018)^24^. (iii) The level of economic development has an important impact on CO_2_ emissions, and the level it is one of the most important factors affecting carbon dioxide emissions (Liu et al. 2022)^25^. This paper adopts per capita GDP to measure the level of economic development.

The definitions of the above variables are collated as shown in Table 1.

**Table 1:** Model variables.

No	Type	Variable	Description
1	Explanatory variable	Carbon emission growth rate	Annual growth rate of carbon dioxide emissions
2	Core explanatory variables	Green technology progress rate	Green technology patent volume growth rate
3	Moderating variables	Energy input share	Coal use in total energy
4	Other explanatory variables	Labour growth rate	Employment growth rate
5		Technology growth rate	Total factor productivity growth rate
6		Capital growth rate	Capital stock growth rate
7	Control variables	Level of government intervention	Fiscal expenditure as a share of GDP
8		Level of marketisation	Degree of marketisation (Fanzang marketisation index)
9		Level of economic development	GDP per capita

### Data sources and descriptive statistics

The basic data for the growth rate of carbon emissions in this paper mainly come from the China Energy Statistics Yearbook (1995–2020), and the data required for the measurement of the rate of green technological progress come from the patent retrieval database of the State Intellectual Property Office. All other variables are obtained from the China Statistical Yearbook(1995–2020), Cathay Pacific Database (1995–2020), etc., totalling 25 years and 30 provinces, cities and autonomous regions resulting in 750 balanced panel data [missing data related to Tibet are deleted]. The descriptive statistics of the relevant variables are shown in Table 2.

**Table 2:** Descriptive statistics.

Variable name	Sample Size	Mean value	Standard deviation	Minimum value	Maximum value
Carbon emission growth rate	750	0.051	0.167	− 1.912	3.314
Green technology progress rate	750	0.207	0.119	− 0.223	0.616
Technology growth rate	750	− 0.026	0.078	− 0.542	0.919
Capital growth rate	750	0.141	0.056	− 0.044	0.371
Labour growth rate	750	0.008	0.229	− 2.206	2.329
Level of government intervention	749	0.192	0.097	0.052	0.643
Level of economic development	749	9.937	0.987	7.625	12.013
Level of marketisation	720	6.058	2.062	1.290	12.000

### Endogeneity and correlation tests

This section discusses possible endogeneity issues. Green technology levels and carbon emissions have a more pronounced causal relationship with each other and therefore have an endogenous link. As seen from Eqs. (21) and (22), the econometric model in this chapter uses the growth rate of carbon emissions as the regression analysis of the rate of progress of green technology level, and the growth rate includes differentials, effectively mitigating the endogeneity problem (Gan et al. 2011)^26^. In addition, this chapter uses the growth rate of green technology patents as an instrumental variable for the rate of green technology progress, whereas green technology patents need to be prepared several years in advance to be applied, and there is no contemporaneous causal relationship between changes in green technology patents and carbon emissions in theory.

From the empirical data and literature, there are significant spillover effects of carbon dioxide emissions and green technology progress; therefore, there may be a forwards or backwards correlation of carbon emissions and green technology progress between provinces and regions, and there may be mutual influences between different provinces and regions in the same period. These influences may likewise be present in other aspects of the socioeconomy. Therefore, this paper finds that there is intergroup heteroskedasticity and intragroup and intergroup autocorrelation through the test of intergroup heteroskedasticity, intergroup contemporaneous correlation and intragroup autocorrelation of the panel data, and the results of the test are shown in Table 3. Therefore, this paper adopts the feasible generalised least squares (FGLS) method, which can adjust for the contemporaneous correlation in the estimation.

**Table 3:** Correlation and heteroscedasticity test of panel data.

Test	Statistic	*p*-value
Intergroup contemporaneous autocorrelation	chi2 (30) = 27,575.85	0.0000
Intragroup autocorrelation test	F(1,29) = 9.436	0.0046
Between-group heteroscedasticity test	chi2(30) = 27,575.85	0.0000

## Analysis of the empirical results

### Analysis of the benchmark results

Table 4 gives the regression results of Eq. (21). Among these results, Model 1 is a simple model without control variables, and Model 2 and Model 3 gradually increase the explanatory variables. This paper takes model 3 as the benchmark model.

**Table 4:** Model results.

Carbon emission growth rate	Model 1	Model 2	Model 3 (Baseline Model)
Green technology progress rate	− 0.065*** (− 41.737)	− 0.064*** (− 7.628)	− 0.060*** (− 19.866)
Technology growth rate		0.073*** (7.769)	0.113*** (27.101)
Capital growth rate		0.148*** (9.538)	0.158*** (12.661)
Labour growth rate		0.020*** (3.624)	0.033*** (19.614)
Level of government intervention			− 0.026*** (− 2.712)
Level of economic development			− 0.031*** (− 10.447)
Level of marketisation			− 0.022*** (− 33.083)
Individual fixed effects	Control	Control	Control
Time fixed effects	Control	Control	Control
N	720	720	720
Constant term	− 0.067*** (− 6.434)	− 0.083*** (− 9.168)	0.087*** (− 8.654)

t-values for regression coefficient estimates in parentheses, ***p < 0.01, **p < 0.05, *p < 0.

As seen from Table 4, the regression results of Models 3 show that the growth rate of the green technology level has a significant negative impact on the growth rate of carbon emissions at the 1% level. The coefficient gradually becomes larger with the increase of control variables, but the overall maintenance coefficient of approximately − 0.06, as seen from the previous theoretical model analysis indicates that $\alpha_{1} = \frac{\alpha }{1 - \alpha } - \lambda = - 0.06 < 0$, corresponding to the theoretical analysis of the situation in Figs. 1 and 2; this implies that as the rate of green technological progress continues to improve, green technological progress raises the increase in the rate of growth of carbon emissions of the scale effect; however, this promotion is smaller than that of the promotion of the rate of growth of carbon emissions of the technical effect in terms of the reduction of the combined effect. Regarding the decrease in the carbon emissions growth rate, there are carbon peak and carbon emission reduction situations, and the empirical results support the theoretical proposition 3, hence it is established.

From the regression results of Models 2–3, it can be seen that the regression coefficients of the capital growth rate, labour growth rate, and technological growth rate of capital and labour are all significantly positive at the 1% level, which means that an increase in the growth rate of capital, labour, and technology promotes an increase in the growth rate of carbon emissions. From Eq. (11), it can be seen that the increase in the growth rate of capital, labour and technology lead to an increase in the growth rate of GDP, thus promoting the increase in the growth rate of carbon emissions due to the scale effect. The empirical results are consistent with the theoretical model.

From the regression results of model (3), it can be seen that the level of government support, the level of economic development and the level of marketisation on the growth rate of carbon emissions are significantly negative at the 1% level, which means that the increase in government support, the continuous development of the economy and the increase in the level of marketisation will contribute to the decrease in the growth rate of carbon emissions.

### Moderating effect test

Table 5 gives the regression results for Eqs. (22). Among these results, model 1 is the benchmark model, and it is consistent with the results of the benchmark model in Table 4. Model 2 is a separate regression result of the energy input share on the growth rate of carbon emissions, and model 3 is a regression result that includes the interaction term of the green technology progress rate and the energy input share, reflecting the moderating role of the energy input share.

**Table 5:** Results of the energy input share test.

Carbon emission growth rate	Model 1 (baseline model)	Model 2	Model 3
Green technology progress rate	− 0.060*** (− 19.866)		− 0.667*** (− 11.62)
Share of energy inputs		0.0514*** (11.397)	0.0256*** (5.46)
Green technology progress rate *Share of energy inputs			0.071*** (11.00)
Technology growth rate	0.113*** (27.101)	0.117*** (26.000)	0.115*** (18.40)
Capital growth rate	0.158*** (12.661)	0.158*** (11.533)	0.172*** (10.38)
Labour growth rate	0.033*** (19.614)	0.032*** (17.778)	0.030*** (10.85)
Level of government support	− 0.026*** (− 2.712)	− 0.0923*** (− 10.313)	− 0.007 (− 0.47)
Level of economic development	− 0.031*** (− 10.447)	− 0.0626*** (− 19.202)	− 0.050*** (− 24.14)
Level of marketisation	− 0.022*** (− 33.083)	− 0.0202*** (− 30.376)	− 0.019*** (− 14.81)
Individual fixed effects	Control	Control	Control
Time fixed effects	Control	Control	Control
N	720	720	720
Constant term	0.087*** (− 8.654)	0.320*** (3.313)	0.392*** (3.91)

As seen from Table 5, the regression results of Model 3 show that under the role of moderating variables, the growth rate of green technology level on the growth rate of carbon emissions are all significantly negative at the 1% level, and there is $\beta_{1} = - \lambda = - 0.667$, so $\lambda = 0.667$ can be calculated from Model 3. From Table 4, $\frac{\alpha }{1 - \alpha } - \lambda = - 0.06$, from which $\alpha = 0.375$ can be calculated. The regression coefficient of the interaction term of the rate of green technological progress and the share of energy inputs on the rate of green technological progress is significantly positive at the 1% level, i.e., $\beta_{2} = 0.071$. This implies that the moderating effect of the energy input share on the scale effect is significantly positive, which is consistent with theoretical proposition 1. The regression coefficient of the share of energy inputs on the growth rate of carbon emissions is significantly positive at the 1% level, implying that a higher share of energy inputs is associated with higher carbon emissions.

In Models 2–3, the regression coefficients for the growth rate of capital, the growth rate of labour, and the technical growth rate of capital and labour are all significantly positive at the 1% level, which is consistent with the results of the benchmark model; the regression coefficients for the level of governmental development, the level of economic development, and the level of marketization are mostly significantly negative at the 1% level, which is consistent with the results of the benchmark model.

### Robustness tests

In this section, the benchmark model of Eq. (19) is tested for robustness by shortening the sample years and adding control variables. First, by selecting samples since 2002 for the regression; second, by drawing on Dong and Wang^27^, foreign direct investment (FDI) is added as a new control variable, and it has an impact on carbon emissions (Shao et al. 2016)^28^ and plays a dual role, which may be either a "pollution halo" effect or a "pollution refuge" effect^29^. Therefore, FDI is included in the benchmark model. The regression results are shown in Table 6, where Model 4 is a robustness test model with shortened sample years and Model 5 and Model 6 is a robustness test model with new control variables. The results of Models 6show that the effect of the green technology progress rate on the growth rate of carbon emissions is still significantly negative at the 1% level, and the models are overall robust.

**Table 6:** Robustness test results.

Carbon emission growth rate	Baseline model	Model 4	Model 5	Model 6
Green Technology Progress Rate	− 0.060*** (− 19.866)	− 0.037*** (− 3.628)	− 0.059*** (− 20.462)	− 0.058*** (− 8.656)
FDI	–	–	0.002 (− 1.127)	
Energy consumption structure	–	–	–	0.0525*** (8.750)
Technology Growth Rate	0.113*** (27.101)	0.066*** (3.609)	0.113*** (28.541)	0. 103*** (1.198)
Capital growth rate	0.158*** (12.661)	0.019 (0.431)	0.158*** (13.008)	0.115*** (5.227)
Labour growth rate	0.033*** (19.614)	0.020*** (2.978)	0.033*** (20.688)	0.029*** (7.250)
Level of Government Intervention	− 0.026*** (− 2.712)	− 0.179*** (− 4.282)	− 0.024*** (− 2.727)	− 0− .031*** (− 0.150)
Level of economic development	− 0.031*** (− 10.447)	− 0.061*** (− 11.815)	− 0.030*** (− 10.090)	− 0.041*** (− 6.833)
Level of marketisation	− 0.022*** (− 33.083)	0.003 (0.882)	− 0.022*** (− 35.403)	− 0.018*** (− 12.426)
Regional effect	Control	Control	Control	Control
Year effect	Control	Control	Control	Control
N	720	570	720	720
Constant term	0.087*** (− 8.654)	0.739*** (6.512)	0.076*** (5.232)	0.128*** (1.805)
$\chi^{2}$	1.6E + 05	4.6E + 04	1.4E + 05	1.4E + 04

t-values for regression coefficient estimates in parentheses, ***p < 0.01, **p < 0.05, *p < 0.

The regression results of model 4 show that when the number of years is shortened (selecting the years since 2002), the coefficient of the green technology progress rate becomes larger and the absolute value decreases, implying that the negative impact of the green technology progress rate on the growth rate of carbon emissions is decreasing, and a possible explanation is that since 2002, the green technology has already been at a higher level, and the impact of the green technology progress on the growth rate of carbon emissions has been decreasing at a marginal rate, so the later the year, the smaller the impact. A possible explanation is that green technology has been at a higher level since 2002, and the impact of green technology progress on the growth rate of carbon emissions is decreasing at the margin, so that the impact decreases in later years. The regression results of model 5 show that the coefficient of the green technology progress rate increases slightly after the addition of the control variable FDI, but the change is not significant, and the model is still robust. However, the effect of FDI on the growth rate of carbon emissions is not significant, which means that FDI and the growth rate of carbon emissions have a weak effect. Model 6 shows that after adding the control variable energy consumption structure. The impact of green technology progress on carbon emissions remains significantly negative. Further validated the robustness of the results.

## Conclusions and policy recommendations

Based on Aghion et al.^10^, this paper constructs a three-sector production model containing capital, labour and energy, gives the theoretical mechanism of the impact of the green technology progress rate on the carbon emission growth rate, and discusses the conditions for carbon peaking and carbon reduction. Through empirical analyses, this paper further investigates the quantitative impact of the green technology growth rate on the carbon emission growth rate and the moderating role of the energy input share, and the empirical results are consistent with the theoretical conclusions.

The theoretical conclusions of this paper show that with the continuous improvement of the rate of green technology progress, green technology progress promotes the increase in the growth rate of carbon emissions through the scale effect; it promotes the reduction of the growth rate of carbon emissions through the technological effect. The technological effect of the rate of green technology progress on the impact of the growth rate of carbon emissions is greater than the scale effect, ultimately affecting the promotion of the growth rate of carbon emissions as it continues to decrease. When the growth rate of carbon emissions is 0, a carbon peak is achieved; when the growth rate of carbon emissions is reduced to negative, carbon emission reduction is achieved. In addition, the share of energy inputs has a positive moderating effect on the scale effect.

The empirical analysis of this paper shows that the comprehensive impact of the rate of progress of green technology level on the growth rate of carbon emissions is significantly negative at the 1% level, and the impact coefficient is approximately -0.06, which reflects that the comprehensive impact of the rate of progress of green technology on the rate of growth of carbon emissions is negative, and the theory of proposition 3 maintains consistency. The green technology progress rate impact on the carbon emissions growth rate is significantly negative at the 1% level through the technology effect, and the impact coefficient is − 0.667; from this, we can calculate the scale effect of the impact coefficient of 0.607, and hence theoretical proposition 2 is consistent. Through the test of the moderating effect, it is found that the moderating effect of the energy input share on the scale effect is significantly positive at the 1% level, which is consistent with theoretical proposition 1.

This paper conducts a robustness test of the baseline model by shortening the sample years and adding control variables, and the results show that the overall model is robust. Considering the heterogeneity of the coefficients of the impact of the rate of green technological progress on the growth rate of carbon emissions in different provinces, this paper conducted a heterogeneity analysis, and the conclusions show that in the pilot provinces of low-carbon policy, the impact of the rate of green technological progress on the growth rate of carbon emissions is insignificant, while for the provinces that are not pilot provinces of low-carbon policy, the impact of the rate of green technological progress on the growth rate of carbon emissions is significantly negative.This may be due to the fact that after the pilot provinces have formulated low-carbon development plans, the adoption and application of green technologies may face a number of challenges, such as the cost of technology conversion, market acceptance, and related policies and regulations. To some extent, this makes the role of green technologies less prominent.

The policy suggestions given in this paper are as follows: the government should increase the support of financial inputs to the level of green technology and at the same time increase and reduce administrative intervention in market behaviour, which can effectively bring into play the subjective initiative of the market main body and truly develop green technology; at the same time, it is necessary to implement differentiated green technology subsidies or carbon emission subsidy policies according to the differences in resource endowment and industrial structure of different provinces or cities. For example, high energy-consuming provinces should reduce government support, while low energy-consuming provinces should increase government support.

Theoretical and practical significance of the article. (1)This paper constructs an endogenous dynamic model of the impact of green technology progress on carbon emissions, which will be a useful addition to the existing environmental economic theory and endogenous theory; (2)By examining the impact of green technological progress on carbon emissions and its mechanism of action, the mechanism of green technological progress on carbon emissions has been further clarified, providing an effective basis for the role of green technology in China's successful realisation of carbon peaks; (3)As China's economy accelerates its transition to green development during the Fourteenth Five-Year Plan period, it is important to objectively identify and clarify the ways and mechanisms by which green technology progress affects the regional environment. It is important to objectively identify and clarify the impact of green technology progress on the regional environment and its mechanism, explore the internal mechanism of the impact of green technology progress on carbon emission reduction, and find a new development path for China to achieve the dual goals of carbon emission reduction and economic growth in the "14th Five-Year Plan" and even in the longer term. It is of great practical significance to establish a green, low-carbon, circular and sustainable modern industrial system.

For space reasons, the article does not analyse the relevant policies. In the next study, consider adding the study of carbon tax policy, green energy subsidy policy, and carbon tax policy to explore the carbon emission reduction effect of related policies. To propose more rationalised policy support for carbon emission reduction.

## Data Availability

The basic data for the growth rate of carbon emissions in this paper mainly come from the China Energy Statistics Yearbook (1995–2020), and the data required for the measurement of the rate of green technological progress come from the patent retrieval database of the State Intellectual Property Office. All other variables are obtained from the China Statistical Yearbook (1995–2020), Cathay Pacific Database (1995–2020), etc.
